# Nursing education challenges and solutions in Sub Saharan Africa: an integrative review

**DOI:** 10.1186/s12912-018-0272-4

**Published:** 2018-01-31

**Authors:** Thokozani Bvumbwe, Ntombifikile Mtshali

**Affiliations:** 1 Faculty of Health Sciences, Mzuzu University, P/ Bag 201, Luwinga, Mzuzu, Malawi; 2 School of Nursing, University of KwaZulu Natal, Durban, 4041 Republic of South Africa

## Abstract

**Background:**

The Lancet Commission and the Global Health Workforce Alliance reported that professional education has generally not kept up the pace of health care challenges. Sub Saharan Africa needs an effective and efficient nursing education system to build an adequate, competent and relevant nursing workforce necessary for the achievement of Sustainable Development Goals. The Plan of Action for Scaling up Quality Nursing and Midwifery Education and Practice for the African Region 2012 - 2022 provided a framework for scale up of nurses and midwives. This integrative review examined literature on nursing education challenges and solutions in Sub Saharan Africa to inform development of a model for improving the quality, quantity and relevance of nursing education at local level.

**Methods:**

A search of PubMed, Medline on EBCSOhost and Google Scholar was conducted using key words: nursing education, challenges, solutions and/ or Africa. Published works from 2012 to 2016 were reviewed to explore reports about challenges and solution in nursing education in Sub Saharan Africa. Full texts of relevant studies were retrieved after reading the tittles and abstracts. Critical appraisal was undertaken and the findings of the relevant studies were analysed using thematic analysis.

**Results:**

Twenty articles and five grey sources were included. Findings of the review generally supports World Health Organisation framework for transformative and scale up of health professions education. Six themes emerged; curriculum reforms, profession regulation, transformative teaching strategies, collaboration and partnership, capacity building and infrastructure and resources. Challenges and solutions in nursing education are common within countries. The review shows that massive investment by development partners is resulting in positive development of nursing education in Sub Saharan Africa. However, strategic leadership, networking and partnership to share expertise and best practices are critical.

**Conclusion:**

Sub Saharan Africa needs more reforms to increase capacity of educators and mentors, responsiveness of curricula, strongly regulatory frameworks, and availability of infrastructure and resources. The review adds to the body of knowledge to enhance efforts of stakeholders in the improvement of the quality, quantity and relevance of nursing education in Sub Saharan Africa.

## Background

Sub Saharan Africa continues to report poor health indicators with challenged health systems due to growing burden of diseases including HIV/ AIDS and non-communicable diseases and a severe shortage of health care workers [1–4]. Shortage of healthcare workers threatens the sustainability of health care systems and negatively affects the achievement of newly launched Sustainable Development Goals (SDGs) in many countries [5, 6]. Sub Saharan Africa is worst hit region with a shortfall of more than 600,000 nurses needed to scale up priority interventions [7]. The Lancet Commission and the Global Health Workforce Alliance reported that professional education has generally not kept up the pace of health care challenges [8–10]. The commission noted challenges with health professional education namely; mismatch of competencies to patient and population needs, poor teamwork, persistent gender stratification of professional status, narrow technical focus without broader contextual understanding, predominantly hospital orientation at the expense of primary care and weak leadership to improve health system performance [11].

There is a global consensus that nurses and midwives constitute the majority of the global health workforce and the largest health care expenditure [8]. Nurses form the universal access point for almost 90% of healthcare users [12, 13]. Considering the significance of nursing workforce within the health care systems, efficient production, successful deployment, and ongoing retention are key to ensuring improvements in the functioning and impact of health care system including ensuring universal health coverage [9, 14]. Efficient production of nurses with relevant competencies remains a critical role of nursing education. Improvements in nursing and midwifery education are recognized as essential in increasing workforce numbers and enhancing the quality of health care and health systems.

Traditionally, nursing and midwifery education has been offered at stand-alone training institutions with more emphasis of production of lower level cadre. Many countries including Malawi, Zambia, Kenya, Zimbabwe still have majority of their nurses at technician level than registered nurse level. However, nursing and midwifery education in Africa has shown signs of development over the past two decades. World Health Organization [15] reported that many countries have now including Masters or Doctoral levels studies in their programs. Nursing colleges in many countries are affiliating their programmes to universities. The Geneva Declaration of the SIDIEF adopted in 2012 urges Francophone countries to introduce university education system for nurses and make undergraduate programme an entry requirement for the nursing profession [16]. Similarly, the Plan of action for Scaling Up Quality Nursing and Midwifery Education and Practice for the African Region 2012 – 2022 provides a framework for WHO member states to improve nursing and midwifery education and training and produce well trained nurses and midwives [17].

However, literature still report lack of necessary competencies among graduates due to lack of strategic leadership to drive transformation [9], unresponsive curricula [18, 19], shortage of nursing faculty and shortage of teaching and learning resources resulting into inadequate productive capacity of training institutions. Globally, nursing education continues to experience underinvestment, static and rigid curriculum, lack of inter-professional preparation of nurses and lack of coordinated collaboration and support from stakeholders.

The Plan of Action for Scaling up Quality Nursing and Midwifery Education and Practice for the African Region 2012 – 2022 provided a framework for scale up of nurses and midwives in the region. The purpose of this study was to review literature on status of nursing education in Sub Saharan Africa to inform development of a model for improvement of quality, quantity and relevance of nursing education at local level.

## Methods

The researchers were informed by the works of Whittemore and Knafl [20] which has five stages; problem identification, literature search, data evaluation, data analysis and presentation.

### Problem identification

The research problem emanated from a Norwegian Church Aid (NCA) funded National Nursing Education Research Conference and anecdotal notes from nursing education stakeholders who identified shortage of nursing workforce and growing concerns of poor quality of nursing care because of inefficiencies in the nursing education system. The review was guided by the following research questions: what is the status of nursing education in Sub Saharan Africa, and what are the solutions or innovations being taken to improve nursing education?

### Literature search

**Fig. 1:**
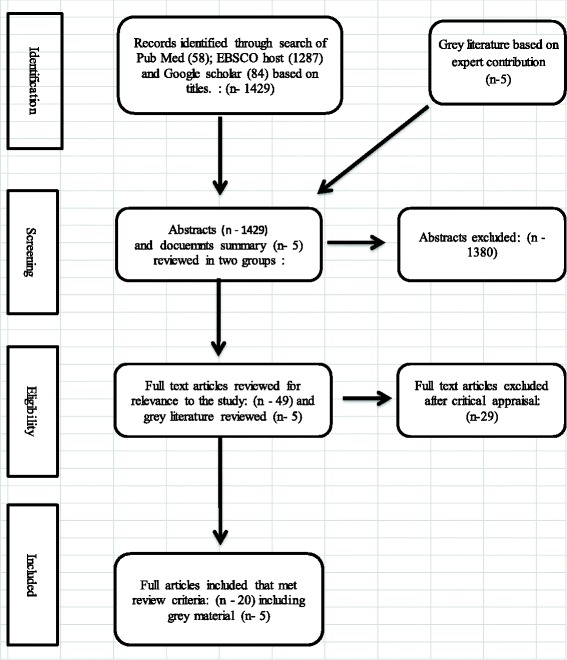
Process of review

A search of PubMed, MEDLINE, Academic search complete, health sources on EBCSOhost and Google scholar was conducted using the key words; nursing education, challenges, solutions, innovations AND Africa. The inclusion criteria for electronic records included primary source and peer reviewed reports on nursing education in Sub Saharan Africa. The review included literature from 2012 to 2016 to capture what has been reported during the period after the Plan of action for Scaling up Quality Nursing and Midwifery Education and Practice for the African Region 2012 – 2022. Peer reviewed records were targeted to ensure integrity of findings because they already have a level of scrutiny. The review also included a search of grey literature as well as extensive consultation with nursing education experts to identify relevant documents. Key stakeholders including nurse educators, nursing education policy makers were individually approached to voluntarily provide material they knew would be relevant including program documents, project reports and progress reports on nursing education. The process of the integrative review is presented in Fig. 1.

### Data evaluation

Records were evaluated for their authenticity, methodological quality and informational value. A structured data extraction and quality appraisal checklist was utilized on each record for information extraction based on the Critical Appraisal Skills programme Checklist. Initially records were selected based on their titles. Abstracts of selected titles were analysed to assess their relevance to the study question. Only abstracts that addressed nursing education challenges and solutions in Africa after 2012 were subjected to a full text review. Full text records that did not meet the appraisal process were excluded from the review. Relevant bibliographies from the identified records were also reviewed.

### Data analysis

Thematic analysis was used to analyse data. Data was extracted and coded into manageable framework. This was then displayed to convert extracted data from individual sources into a display that assembles around particular variables or subgroups. Extracted data was compared item by item so that similar data was categorised and grouped together. An iterative process of examining data displays to facilitate distinction of patterns, themes, variations and relationships was done using a comparison method. Conclusions were then drawn from the data. To ensure reliability of the review, the articles and the appraisal forms were independently reviewed by two colleagues. Findings from the researchers and the independent evaluators were compared and discussions were held to reach an agreement.

## Results

The search conducted yielded a total of 1434 records. One thousand two hundred and eighty seven (1287) records were obtained from EBSCOhost database, 58 records from PubMed and 84 from Google scholar and 5 grey records obtained from experts. One thousand three hundred and eighty (1380) abstracts were excluded either because they addressed nursing education outside Sub Saharan Africa or addressed education of health professions in general. Twenty nine (29) full text reports were excluded from the study because they neither addressed the challenges nor the solutions of nursing education. Majority of the records addressed perceptions and experiences of nursing students with the nursing training. Finally, twenty (20) records and five (5) grey material were finally included in the review (Table 1).

**Table 1:** Summary of records reviewed

Author and year	Country	Tittle	Contribution to nursing education
Middleton, L., Howard, A, Dohrn, J, Von Zinkernagel, D. et al., 2014	Sub Saharan	The Nursing Education Partnership Initiative (NEPI): Innovations in nursing and midwifery education.	Building on existing country leadership and government planning to increase the number and competencies of new nurses entering the profession. Country-level leadership, support for faculty workforce development and key educational processes and practices, and scaling up of proven educational methods and interventions at nursing educational institutions are key to ensuring sustainability and will help to achieve priority health goals for the countries of this region.
Mthembu, S., Mtshali, N., Frantz, J. 2014	South Africa	Contextual determinants for community-based learning programmes in nursing education in South Africa	Community Based Learning has emerged as a political instrument that has influenced not only a change in the country’s health system but has responded to the needs of the South African population at large
Goosby, E.P, von Zinkernagel, D. 2014	Sub Saharan	The Medical and Nursing Education Partnership Initiatives.	The MEPI and NEPI focus on strengthening learning institutions is central to the vision for expanding the pool of health professionals to meet the full range of a country’s health needs. A robust network of exchange between education institutions and training facilities, both within and across countries, is transforming the quality of medical and nursing education and augmenting a platform for research opportunities for faculty and clinicians, which also serves as an incentive to retain professionals in the country.
Jooste, K. & Jasper, M. 2012	South Africa	A South African perspective: current position and challenges in health care service management and education in nursing.	Different role players are involved in critical issues regarding the management and education of nursing in South Africa. Nurse managers are central to the success of service redesign, delivery and education. Nurse managers need to influence policy decisions regarding nursing service design and delivery, and the education required to prepare the next generation of practitioners for these new services.
Armstrong, S. & Rispel, L. 2015	South Africa	Social accountability and nursing education in South Africa	Social accountability, which is an essential component of transformative education, necessitates that attention be paid to the issues of governance, responsive curricula, educator preparedness, and appropriate student recruitment and selection
Adejumo, O., Fakude, L. & Linda, N., 2014	South Africa	Revisiting innovative approaches to teaching and learning in nursing programmes: Educators’ experiences with the use of a case-based teaching approach at a nursing school.	Concerns raised included issues about the facilitation role of the teacher; the role of the student; curriculum alignment; assessment methods; and the role of the environment in case-based teaching and learning settings.
Marchi-Alves, L.M., Ventura, A., Trevizan, M.A., Mazzo, A. et al. 2013	Angola	Challenges for nursing education in Angola: the perception of nurse leaders affiliated with professional education institutions.	Lack of infrastructure, absence of trained human resources experts, bureaucratic problems to regularize the schools and lack of material resources hinder improvements. Public health education policies need to be established in Angola, including action guidelines that permit effective nursing activities. Professional education institutions need further regularizations and nurses need to be acknowledged as key elements for the qualitative enhancement of health services in the country.
Mutea, N. & Cullen, D., 2012	Kenya	Kenya and distance education: A model to advance graduate nursing.	A collaborative model is presented as a potential solution to advance graduate nursing. Four major constituents are identified including hospitals and agencies, communities of interest, Kenyan universities and international education partners. Each has a part to play including contributions to information, communication of opinion and expertise, money and support, infrastructure and in-kind resources. Distance education is cost-effective and will help in building capacity at various levels of nursing including leadership in clinical practice, teaching, administration and research.
McCarthy, C.F., Voss, J., Salmon, M.E., Gross, J.M., Kelley, M.A. & Riley, P.L, 2013	East, Central, and Southern Africa	Nursing and midwifery regulatory reform in East, Central, and Southern Africa: a survey of key stakeholders.	Information regarding effectively engaging leaders in regulatory reform by clarifying their roles, responsibilities, and activities regarding regulation overall as well as their specific perspectives on task shifting and pre-service reform.
Daniels, F.M., Linda, N.S., Bimray, P. & Sharps, P., 2014	South Africa	Effect of increased student enrolment for a Bachelor of Nursing programme on health care service delivery.	The changing learning environment, competition for learning opportunities and limitations in terms of clinical support posed challenges for professional nurses to perform their multifaceted role which includes clinical teaching and mentoring, and affected service delivery. Therefore, careful planning of students’ learning experiences in both theory and practice is imperative to ensure that teaching and learning and service delivery are not negatively affected
Wilson, L.L., Somerall, D., Theus, L., Rankin, S., Ngoma, C. & Chimwaza, A., 2014	Malawi, Zambia,	Enhancing global health and education in Malawi, Zambia, and the United States through an inter-professional global health exchange program	Program promoted inter-professional and cross-cultural understanding; fostered development of long-term sustainable partnerships between health professionals and educators in Zambia and the US; and created increased awareness and use of resources for global health education
Livingston P., Bailey J, Ntakiyiruta G, Mukwesi C, Whynot, S. & Brindley, P., 2014	Rwanda	Development of a simulation and skills centre in East Africa: a Rwandan-Canadian partnership.	Developed an adaptable model for simulation and skills centre development in low-resources settings.
Jacob, S., Holman, J., Msolomba, R., Wasili, R., Langdon, F., Levine, R., Mondiwa, M., Bateganya, M. & MacLachlan, E, 2015	Malawi	Using a task analysis to strengthen nursing and midwifery pre-service education in Malawi	Using task analysis, the identified gaps in clinical training and faculty supervision of students. The task analysis provided a robust approach to curriculum revision through identifying key content gaps. Other countries might consider adopting this approach to improving the content and relevancy of nursing and midwifery syllabi and curricula.
Kurth, A., Jacobs, S., Squires, A., Sliney, A., Davis, Stalls. & Portillo, C. 2016	Rwanda	Investing in nurses is a prerequisite for ensuring universal health coverage	The World Health Organization endorses task sharing to ensure universal health coverage in HIV and maternal health, which requires an investment in nursing education, retention, and professional growth opportunities.
Mtshali, N.G. & Gwele, N.S., 2016	South Africa	Community-based nursing education in South Africa: A grounded-middle range theory	The input from the community enhances the relevance of the curriculum to the priority needs of the surrounding community. It also ensures that the CBE curriculum is dynamic and is based on the present, because of the changes taking place in the community.
Botma, Y., 2014	Botswana	How a monster became a princess: Curriculum development.	Changing content driven curriculum to competence based curriculum improved training on nurses with a primary care focus
Bell, S.A., Rominski, S., Bam,V., Donkor, E. & Lori, J., 2013	Ghana	An analysis of nursing education in Ghana: Priorities for scaling-up the nursing workforce	Faculty and infrastructure shortages are common issues in nursing education and workforce expansion, these issues arecompounded by high rates of preventable disease and injury.
Kiarie, J., Farquhar, C., Redfield, R., Bosire, K., Nduti., Mwanda, W., M’lmunya, J. & Kibwage, I., 2015	Kenya	Strengthening health systems by integrating health care, medical education, and research: University of Nairobi experience.	The study suggested innovation building capacity of healthcare workers and students through the eBNM program. Students felt they had more opportunities to practice clinical skills, closer mentoring, and closer interactions with patients at the at the non-tertiary facilities than at the tertiary hospital. Health workers at the non-tertiary hospitals also reported improved quality of patient care, increased job satisfaction, and greater interest in research. County hospitals have retained employees and the nurses are upgrading their skills without losing income.
Blaauw, D., Ditlopo, P. & Rispel, L. 2014	South Africa	Nursing education reform in South Africa – lessons from a policy analysis study	The study found significant weaknesses in the policy capacity of the main institutions responsible for the leadership and governance of nursing in South Africa, which will need to be addressed if important nursing education reforms are to be realised
Appiagyei, A.A. Kiriinya, R.N., Gross, J.M., Wambua, D.N., Oywer, E.O., Kamenju, A.K., Higgins, M.K., Riley, P.L. & Rogers, M.F. 2014	Kenya	Informing the scale-up of Kenya’s nursing workforce: a mixed methods study of factors affecting pre-service training capacity and production	To scale-up the nursing workforce in Kenya, concurrent investments in expanding the number of student nurse clinical placement sites, utilizing alternate forms of skills training, hiring more faculty and clinical instructors, and expanding the dormitory and classroom space to accommodate new students are needed to ensure that increases in student enrolment are not at the cost of quality nursing education.

Six themes emerged from the review namely; curriculum reforms, professional regulation, transformative teaching strategies, collaboration and partnership, capacity building and infrastructure and resources.

### Theme 1: Curriculum reforms

Health care reforms alter the environment in which nurses and other health care workers practice. The review found that in Sub Saharan Africa, emphasis on primary care, emerging of new health challenges, increasing burden of disease significantly demand need for curriculum reforms [21–23]. Mthembu et al. [22] reported that reforms in curricula are necessary to ensure that nursing education produces graduates who influence the quality of the health care system and are relevant to the needs of the population.

Mtshali and Gwele [21] developed a middle range model that would guide the practice of community based education in basic nursing education in South Africa. This curriculum reform in nursing education reported community based education as relevant, responsive education and education for social justice that consciously and deliberately socialize health delivery towards primary health care. We also found that countries within Sub Saharan Africa are prioritizing curriculum reforms from content driven curriculum to competence based curriculum that are responsive to the primary health care [23]. We also found that prevalence of chronic illnesses and co-morbidities that are emerging demands an inter-professional approach and collaboration to manage health [24].

### Theme 2: Profession regulation

Within the changing face of nursing education, key issues in Sub Saharan Africa related to professional regulation include notions of specialist and advanced practice, accountability and autonomy, competence, supervision, continuing education and delegation [25]. McCarthy et al. [25] highlighted that nursing education should complement, advocate and lend technical support to regulatory bodies as regulatory frameworks change due to health care adaptation. The review highlighted that strengthening nursing councils and professional associations helps to improve regulatory activities. Licensure, accreditation, continuing professional development, and scope of practice influence the quality of care provided by the nursing workforce as well as pre-service education program [26].

### Theme 3: Transformative teaching strategies

Appiagyei et al. [27] observed that congestions of students in clinical sites will demand innovative ways to train the growing numbers. As student populations and methods of learning continue to increase in diversity, nurse educators and administrators must be flexible and responsive with effective and innovative solutions to complex market demands. The review found that innovative approaches to teaching and learning are significant for teaching future professions [28]. The complexity of health care demands requires critical thinking as well as competencies that are relevant to these demands. Thus demands for the use of innovative methods of teaching that engage students as active learners, continue to grow. Technology facilitates students’ exposure to clinical scenarios that they would normally not encounter in their normal clinical setting [29–31].

### Theme 4: Collaboration and partnerships

We found that the complexity of healthcare demands and practice environment require a coordinated and collaborative approach to training of health professionals including nurses. However, the review show that nurses’ engagement in policy making is still complex and contested [32]. Evidence based practice requires decisions about healthcare based on best available, current, valid and relevant evidence. Goosby and von Zinkernagel [33] highlighted that partnerships form a strong foundation for planning and delivery of evidence based health services.

We found that the changing learning environment, competition for learning opportunities and limitations in terms of clinical support pose challenges for professional nurses to perform their multifaceted role [34]. Professional nurses play dual roles of service delivery and clinical teaching and mentoring. Careful planning of students learning experiences is imperative to ensure that students get maximum benefits from their training. Middleton et al. [35] reported that current reforms in nursing education demands sharing of knowledge and information. National and regional networks increase opportunities for sharing best practice in nursing.

### Themes 5: Capacity building

Challenges facing nursing education in Sub Saharan Africa demands strong committed leadership to see mutual goals and strategic contribution and effective use of resources [36]. Building management capacity of nursing training institutions is one strategy that various countries have put in place to ensure adequate and quality education of nurses to strengthen their weak healthcare system. Nursing education operates within a complex environment which has to constantly be put under vigorous evaluation in order to encourage innovations that will allow flexibility and effective advancement. ICAP through the Global Nurse capacity Building Program (GNCBP) in six Sub Saharan African countries embarked on building capacity of faculty in nursing education [35, 37].

The complex environment in nursing education demands that academic managers, leaders and players continually monitor the ever- evolving, complex system of the professional tripartite: nursing education, research and practice. Nursing leaders have been challenged to spearhead a successful translation of scientific knowledge that links nursing education to professional practice in the delivery of high quality health care. Jooste and Jasper [38] reported that nurse managers need to influence policy decisions regarding nursing services design and delivery.

Capacity building also surrounds around the issue of faculty ability to prepare a generation of relevant graduates [35, 37]. The review noted that to improve the quality and quantity of nursing, it is necessary to increase the number of nursing faculty and clinical educators who have both expertise in nursing care and commensurate education.

### Theme 6: Infrastructure and resources

We found that lack of infrastructure, absence of trained human resources experts and lack of material resources posed big challenges to nursing education among Sub Saharan African countries [39, 40]. With the continuing growth in the world’s population and the growing disease burden, there is a critical need for increased numbers of qualified health-care personnel and increasingly more efficient healthcare systems. The review shows that the shortage of nurses and midwives has led to increased student enrolment in most countries [34, 39]. Increase in student enrolment has resulted in a strained clinical learning environment, competition for learning opportunities among students [35]. We found that infrastructure investment will facilitate better quality education [39]. The strategy involved providing infrastructure development for the nursing training institutions to accommodate more students and to provide student training fees.

## Discussion

This integrative review has attempted to synthesize relevant published work on nursing education in Africa and made recommendations towards improving the quality, quantity and relevance of nursing education in Su Saharan Africa. Despite the reported severe shortage and maldistribution of nurses between and within countries, nurses remain the single largest available group of available health workers. This positions nurses to be global leaders in driving the quality of health care delivery especially in low –resource settings [41]. Numerous literature report a very significant relationship between nursing and patient safety, patient satisfaction and quality care [42–44]. However, nursing education programs have failed to produce the required graduates who are responsive to the local health policies and programs [45] and the needs of the health care users [46–48].

### Curriculum reforms

A curriculum is at the heart of every educational enterprise [49]. The change in the global health care landscape is putting much pressure for reforms on how the health care workforce practices. Forbes and Hickey [50] highlighted four themes within the need for curriculum reform namely incorporating safety and quality in nursing education, re-designing conceptual frameworks, strategies to address content laden curricula and teaching using alternative pedagogies. Jacobs et al. [51] used a task analysis to reform curriculum identified gaps in clinical teaching and faculty supervision. Task analysis provided a robust approach to curriculum revising through identifying content gaps. The healthcare system in Sub Saharan Africa is getting more complex. Demand for adequate and quality teamwork among stakeholders in increasing. Approaches that transform systems and encourage the move away from the traditional focus on tertiary care hospitals to initiatives that foster community engagement are needed.

Nursing plays a key role in the coordination and integration of care and services from other providers [52]. Reforms that embrace inter-professional education will help to remove challenges that come about between professions and this facilitates coordinated care [53]. Liaw et al. [54] supports that inter-professional learning is essential to enhance team collaboration and communication necessary for patient safety and effective healthcare. Therefore, curriculum reform that enhances relevance of nursing programs within multidisciplinary teams and inter-professional collaboration is very significant in Sub Saharan Africa.

### Profession regulation

Nursing as a profession has a unique system of rules an principles to regulate its members and demonstrate its responsibility to society [55]. The changing healthcare landscape such as an ageing society, double burden of disease with an emerging burden of non-communicable diseases, coupled with shortage of nursing specialist cadres in remote areas, the gap between healthcare supply and demand, task shifting and the perception that nurses and midwives do not work to their full potential, is necessitating changes in the scope of practice frameworks [56, 57].

International Council of Nurses [58] identified fundamental responsibilities of nurses to promote health, prevent illness, restore health and alleviate suffering. These responsibilities deal with challenges of determining the scope of professional nursing practice. Schluter et al. [59] argue that the changing pattern of healthcare delivery ultimately leads to a reconsideration on how nurses manage their work in line with associated legislation. Many countries in Sub Saharan Africa have regulatory nursing bodies which are responsible for developing and implementing a regulatory framework. However, several study across the region reported that regulatory bodies do not always have sufficient capacity and resources to face reform demands [9, 60].

### Transformative teaching strategies

Many health professional training programs have mostly maintained classroom teaching. These short term lectures and seminars have proved not to be effective in diversifying skills md competencies of health workers including nurses [9]. The review indicates that Sub Saharan Countries have increased healthcare training intakes to solve the shortage of human resource crisis. This has led to congestion of students in clinical sites. Increased intakes demand innovative ways to train these growing numbers. King [61] reported that further expansion of distance education is an innovative and cost effective way of advancing nursing and midwifery in Sub Saharan Africa.

Nurse educators need to examine what they do in and out of classroom to continue to be effective, current and relevant. With the focus on community health demands, training initiatives should prioritise the acquisition of competencies through sustained mentorship and supervision, simulation rather than through ad hoc short term lectures and seminars [62, 63].

### Collaboration and partnerships

In some instances a disjuncture between nursing leadership and front line nurses has been reported [64, 65]. World Health Organization [66] advocates that educational and training institutions should foster and enhance the relational activity, interaction and planning between education, health and other sectors. Both academia and practice have the overall goal of attaining optimal health for their countries.

Careful planning of students learning experiences between academia and practice is imperative to ensure that students get maximum benefits from their training. Current reforms in nursing education demands sharing of knowledge and information. Partnerships between academia and practice can contribute significantly towards a vibrant healthcare system. Effective academic practice partnerships can reduce the theory practice-gap thereby improving patient safety, reducing medical errors, strengthening practice setting and cushioning faculty shortage [67, 68]. Academia and practice are dissimilar but share values regarding nursing education. An academic practice partnership can therefore be best understood from a view point where the academic and the practice players come together and work collaboratively for a common goal [69]. Implementation of the shared goal should involve specific responsibilities for the educators, hospital administrators, students and the nurse practitioners through a systems approach [70].

Academic practice partnerships provide a platform for partners to capitalize on each other’s expertise. This also improves access to a broader array of clinical experiences for students. Students receive adequate clinical support that is blended with expertise from both academic knowledge and practice competencies. Evidence supports that mentorship provided by clinical personnel is critical to students’ training outcomes [71]. Quality clinical practice outcome is dependent on preparation and willingness of practice partners. Narasimhan et al. [72] noted that lack of mutual planning results in over-expenditure on unimportant activities within the effort to improve quality of nursing education.

### Capacity building

World Health Organization [60] highlighted that significant investment will be required to strengthen educational infrastructure, faculty and staff development, curriculum review and clinical instruction. To improve the quality and quantity of nursing, it is necessary to increase the number of nursing faculty and clinical educators who have both expertise in nursing care and commensurate education. Evidence from South Africa indicated that the education and training system for the health sector has not grown sufficiently to meet health needs and health system requirements. This is in part due to lack of integrated planning between the health and education sectors on the development of health professionals in relation to health care need, and inadequate financing mechanisms for health professional development.

### Infrastructure and resources

Health worker shortage in Sub Saharan Africa is a result of many causes which include past investment shortfalls in pre- service training, international migration, career changes among health workers, premature retirement and morbidity and mortality [7]. Experience shows that despite the increased enrolment in most countries, the number of quality clinical environment has remained the same. The review has highlighted that clinical learning environment is been strained. There is need to deliberately invest in infrastructure both at training institutions and practice levels.

#### Strengths and limitations of the study

This review shares useful information for improving nursing education among stakeholders worldwide. The major limitation of the study was that it only focused on literature from Sub Saharan Africa.

#### Implication for practice

Nursing and midwifery remains the backbone of the healthcare system worldwide. Continued efforts to improve training of nurses and midwives will ensure improved health outcomes. Effective nursing education will ensure provision of competence based practice among nurses. More research on nursing education will help to improve practice standards in nursing and midwifery.

## Conclusion

The integrated review highlights that majority of countries within Sub Saharan Africa are experiencing common challenges ranging from strained training institutions due to increased enrolments, inadequate faculty capacity, lack of infrastructure and resources, high demand for clinical training sites. To ensure improved quality and quantity of production, developmental partners have increased allocation of financial resources for infrastructure and teaching and learning material. Efforts are being done to expand number of clinical sites, build faculty capacity and increase collaboration with clinical institutions for clinical instructors and mentors. Curriculum reforms are being implemented to reposition the nursing workforce for a competence based approach, community based education and inter-professional training. Clinical simulation and technology based teaching strategies are on the increase to accommodate the demands for new ways of teaching and learning.

## Abbreviations

HIV/AIDS: Acquired Immunodeficiency Syndrome/ Human Immunodeficiency Virus
NCA: Norwegian Church Aid
SDGs: Sustainable Development Goals
